# The evolution of social learning mechanisms and cultural phenomena in group foragers

**DOI:** 10.1186/s12862-017-0889-z

**Published:** 2017-02-10

**Authors:** Daniel J. van der Post, Mathias Franz, Kevin N. Laland

**Affiliations:** 1 Center for Social Learning and Cognitive Evolution, School of Biology, St Andrews University, Harold Mitchell Building, St Andrews, KY16 9TH UK; 2 Leibniz Institute for Zoo and Wildlife Research, Alfred-Kowalke-Straße 17, Berlin, 10315 Germany

**Keywords:** Multi-scale approach, Agent-based model, Mechanism specificity, Traditions, Cumulative culture, Self-organization

## Abstract

**Background:**

Advanced cognitive abilities are widely thought to underpin cultural traditions and cumulative cultural change. In contrast, recent simulation models have found that basic social influences on learning suffice to support both cultural phenomena. In the present study we test the predictions of these models in the context of skill learning, in a model with stochastic demographics, variable group sizes, and evolved parameter values, exploring the cultural ramifications of three different social learning mechanisms.

**Results:**

Our results show that that simple forms of social learning such as local enhancement, can generate traditional differences in the context of skill learning. In contrast, we find cumulative cultural change is supported by observational learning, but not local or stimulus enhancement, which supports the idea that advanced cognitive abilities are important for generating this cultural phenomenon in the context of skill learning.

**Conclusions:**

Our results help to explain the observation that animal cultures are widespread, but cumulative cultural change might be rare.

**Electronic supplementary material:**

The online version of this article (doi:10.1186/s12862-017-0889-z) contains supplementary material, which is available to authorized users.

## Background

Individuals in various animal species develop group specific behavioral habits through learning and cultural transmission [1]. In many cases such behavior is directly related to diet preferences and extractive foraging, and is thought to enhance survival and reproductive success [2]. In humans, cultural inheritance can also enable individuals to acquire complex skills and knowledge that would not be possible within the lifetime of a single individual [3, 4]. Such culturally defined phenotypes are known to have a considerable impact on the evolutionary process [5].

Here we use the term ‘cultural phenomena’ to refer to processes whereby behaviors are inherited across generations via social influences on learning. We focus on (1) *traditions*, which are group-specific behavioral patterns that remain stable over time, and (2) *cumulative cultural change*, which describes cultural change across generations that allows individuals to achieve phenotypes that they could not achieve within their lifetime through asocial learning. Traditions have been identified in various animal species, including fish [6, 7], capuchin monkeys [8], great apes [9–11] and cetaceans [12]. In contrast, cumulative cultural change is generally considered in the context of technical skills and is widely considered to be uniquely human [4, 13].

Explanations for the prevalence of both these cultural phenomena across the animal kingdom have focused on social learning mechanisms and the cognition that these are assumed to entail. The term ‘social learning mechanism’ (henceforth SLM) relates to the kinds of cues to which individuals pay attention when learning from conspecifics [14, 15]. When considering inheritance of traditions, some researchers have emphasized that cognitively demanding forms of social learning would be required to maintain the fidelity and adaptedness of traditions [16–20]. Moreover, it is generally accepted that the key to generating cumulative cultural change is transmission fidelity of learned skills, and the cognitively demanding forms of social learning on which the latter is thought to rely [4, 13].

In contrast, results from multi-scale simulation models suggest that simple SLMs, like local and stimulus enhancement, are sufficient for generating traditional differences between groups and cumulative cultural change of diet repertoires in the context of learning what to eat [21–23]. This result extends the concept of cumulative culture to ‘non-technical’ learning contexts, although it should be noted that the cumulative cultural change found in the models [21, 22, 25] can be characterized as bounded, in the sense that it is restricted to a fixed set of existing opportunities in the environment. This contrasts with the apparent open-ended cumulative cultural processes mentioned above, often with increases in behavioral complexity, a characteristic of humans, and implies that researchers should distinguish between different kinds of cumulative cultural process. In any case, the simulation results generate the hypothesis that special cognitively sophisticated forms of SLM are not necessary for generating traditional differences and cumulative cultural change.

In the present study, we investigate whether this hypothesis also holds in the context of skill learning, the more intuitive context in which to consider cumulative cultural change. It may be fairly straightforward to generate cultural phenomena in relatively simple learning contexts, such as diet learning, since high fidelity copying only concerns what kind of resource is interacted with. For skill learning, high fidelity copying would involve both what resource is interacted with and how it is interacted with. Whether simple SLMs suffice to generate cultural phenomena in this context is therefore uncertain.

To evaluate whether different SLMs can support traditional differences between groups and cumulative cultural change in the context of skill learning, we study group foragers that learn what to eat and develop skills in order to process resources, and in the process consider three SLMs. Local enhancement (LE), where an animal is more likely to interact with, and learn about, objects at a particular location following observation of other animals at that location [15], is implemented as arising as a byproduct of grouping [24]. Following van der Post et al. [22], stimulus enhancement (SE) is implemented as an increased probability to choose a given resource type having observed another forager eating that resource type, an implementation that closely follows its definition [15]. Finally, observational learning (OL), which is a general term that represents a number of SLMs, including imitation and emulation [15], following Franz and Matthews [23], is implemented as a gain in skill that is proportional to the difference in skill between an observer and a demonstrator. Of these three SLMs, only OL affects skill learning directly. SE and LE could in principle lead to increases in skill level, but only indirectly. Such indirect increases in skill levels could occur if LE or SE lead to reduced repertoire diversity and enable foragers to spend their limited skill development time on fewer resources [26].

While we test a hypothesis generated by previous theory, our study is not simply an extension of previous models. Here we directly contrast multiple SLMs in a relatively complex model with a large parameter space. To facilitate the exploration of parameter space, we use evolved parameter values for behavioural and learning parameters based on van der Post et al. [26]. In this way parameters are optimized relative to foraging success, and the different SLMs are compared using parameters that derive from this standardized criterion. The earlier models did not use evolved parameters.

In order to include evolved parameters in our model, we included the relatively natural assumptions of dynamic group sizes and stochastic birth-death processes in populations with multiple groups [26], as opposed to the fixed group sizes and regularized birth-death processes in simulations with only one or two groups as assumed in earlier models [21–25]. However, since we change the learning context and population level assumptions, and now include evolved parameters, we will be unable to pinpoint the exact cause of any differences in the results we find relative to earlier models. Nevertheless, despite these limitations, our approach is particularly suitable to assess whether simple SLMs are sufficient to generate traditions and cumulative cultural change in the context of skill learning.

Drawing on previous work [21, 24], we focus on cohesive grouping and environments with patchy resources where each patch has multiple resource types. This provides an empirically relevant context for primates and other social learning species, and is the context in which LE was found to generate both cultural phenomena [21]. In addition, previous work has established that protracted learning (as opposed to instantaneous learning) is a prerequisite for both traditions and cumulative culture [24]. If learning is protracted it becomes susceptible to stochastic variation in sampling frequencies, leading to arbitrary differences in the evaluation of, and preferences for, different resource types. This generates a positive feedback where more familiar resources are more favourably evaluated and hence more often chosen [21, 23, 24]. As a result, in diverse environments, learning can lead to idiosyncratic sub-optimal behavioral repertoires (i.e. local attractors in learning space that are history-defined and self-reinforcing). Social learning through LE and SE causes behavioral repertoires and familiarity biases to be shared amongst group members, thereby leading to the emergence of traditions [21–24]. Here, in the context of skill learning, we vary the protractedness of skill learning by varying the ‘task difficulty’ of resources in the environment.

Based on the above mentioned theory we address the following questions: (1) *Do all the SLMs tested generate traditional differences?* (2) *Do all the SLMs tested generate cumulative cultural change?* (3) *Does task difficulty enhance cultural phenomena?* Here we expect the magnitude of traditional differences to increase with greater task difficulty when learning is more protracted, and that cumulative cultural change will occur when within-lifetime optimization is increasingly limited, which should occur with greater task difficulty; (4) *Do SE and OL enhance traditional differences and cumulative culture?* Compared to LE, we expect that traditional differences will be enhanced by SE, because SE enhances within-group similarity [22], and predict that OL will have the same effect. We also expect SE to enhance the cumulative cultural process [22], and in particular, OL, which leads to direct increases in skill levels, is expected to generate cumulative cultural change of large magnitude; (5) *Do cumulative cultural increases in skill level and repertoire quality contribute to energy intake?* Next to increases in repertoire quality [21] we expect increases in skill to contribute to cumulative cultural increases in energy intake. While only OL affects skill learning directly, SE and LE could in principle lead to increases in skill level indirectly. This can happen if LE or SE lead to reduced repertoire diversity and enable foragers to spend their limited skill development time on fewer resources [26].

## Methods

Our model is an event-based, individual-based model with a spatially-explicit environment and is freely available at https://bitbucket.org/dvanderpost/aapjes_bmc_eb_2016_b. The key design feature of the model is that we define behavioral decision making and the outcome of behavioral events, including learning, at a local spatio-temporal scale. We then study the meso- and macro-scale consequences of that local behavior to establish the mapping between different mechanisms at a local scale and information processing and payoffs at a larger scale. While the model is formulated ‘keeping primates in mind’, and a large number of parameter values are based on estimates of natural primate systems, we expect our conclusions to generalize to other animal taxa, particularly those with similar movement patterns and repertoire sizes. The model is based on previous models of learning in group foragers [21, 22, 24], but now includes skill learning, dynamic populations and group sizes, and evolving parameters. Increments in skill arise through asocial learning or through observational learning (a form of social learning).

The following model description is limited to those aspects needed to gain a reasonable understanding of the results, with key parameters listed in Table 1. For further details see Section 1 in Additional file 1.

**Table 1:** List of key parameters and variables

Name	Description	Values
*R*	Number of resources species	250
*Q*_*r*_	Maximum energy reward of resource type *r*	*N*(0.1,0.1)
*H*_*r*_	Practice time needed before obtaining half the maximal reward	0.1..10
*S*_*r*_	Scalar for the sigmoid function describing how rewards increase with practice	1..4
*EC*	Rate at which resource types are replaced by new types	0..*R* types per year
*N*	Population size	100
*G*	Maximum number of foragers in a group	20
COPY_SPACE	Distance at which foragers can observe what their neighbors are doing	20
*K*	Effectiveness of observational learning	0.1
*M*	Maximal time to process and consume a resource item	1 min
*a*_*ir*_	Expected reward forager *i* has for resource type *r*	any
*a*_*ie*_	Expected quality of the environment of forager *i*	any
*e*_*ir*_	Energy reward forager *i* obtains from resource type *r*	any
*c*_*ir*_	Certainty of forager *i* about reward obtained from resource type *r*	0.. inf
*s*_*ir*_	Skill of forager *i* for processing resource type *r*	0..1
*t*_*ir*_	Experience (total time) forager *i* as processing resource type *r*	0.. inf
*λ*_*i*_	Reinforcement learning rate	0..1
*ε*_*i*_	Exploration rate	0..1
*γ*_*i*_	Stimulus enhancement	0..1
*ω*_*i*_	Probability to OBSERVE neighbor	0..1
*τ*_*i*_	Duration of OBSERVE	0.01..1 min
*ϕ*_*i*_	Update parameter for *a*_*ie*_	0..1
*δ*_*i*_	MOVE distance of forager *i*	0.. inf

Upper case letters and names: invariant parameters that do not change during simulations. Lower case letters: (learning) variables that change during a forager’s lifetime. Greek letters: parameters that can evolve but are invariant during a forager’s lifetime. Subscripts: *i*= forager identity; *r*= resource type

### Model overview

We first give a short overview of the model, followed by further details.

**Fig. 1:**
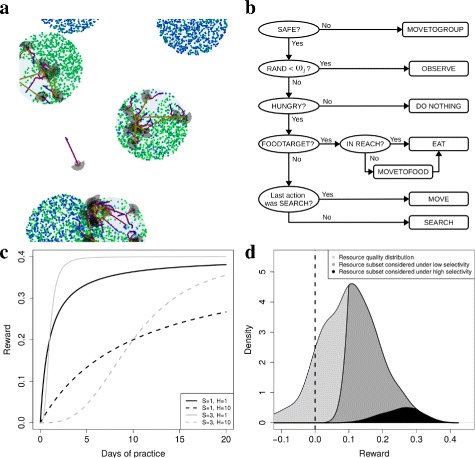
Model details. **a** Simulation snapshot. Each forager is indicated by a SEARCH area (*gray semi-circle*), REACH (*gray circle*) and a movement trajectory (*red to blue line*). When a foragers observes another forager the foragers are connected by an olive-green line. For illustration purposes, the resource items are shown as colored circles, and patches by a larger gray circles. Each patch can be assumed to be a distinct patch type, with unique resource types (different colours within a patch). **b** Illustration of decision-making algorithm. Rectangles are actions and ellipses are decision-making points. After completing one of the actions at the right hand side, all foragers start the decision-making process at the top left (SAFE?). RAND is a random number between 0 and 1, and *ω*_*i*_ is the probability to do OBSERVE. MOVETOFOOD is always followed by EAT. MOVE consists of at many 1 meter steps to complete a distance of *δ*_*i*_. **c** Illustration of how rewards *e*_*ir*_ change with time spent practicing that skill for different resource types (Eq. 5): resources for which not much practice is needed (*solid lines*, low *H*) and those for which a lot of practice is need (*dashed line*, high *H*); and resources for which rewards increase fast immediately (*black lines*, low *S*) and those for which they increase slowly initially (*gray lines*, high *S*). **d** Illustration of how selectivity (Eq. 1) affects which subset of resources are chosen: overall resource quality distribution given by *N*(0.1,0.1) (*light gray*) and subsets chosen when selectivity is low (*dark gray*, *a*_*ie*_=0.1) and high (*black*, *a*_*ie*_=0.3), given *σ*_*i*_=5 and assuming the forager knows all resources perfectly


**Entities:** The model is composed of groups of foragers and patches made up of resource items, which are situated in continuous space (Fig. 1 a and Additional file 2).

Additional file 2: Video showing group foragers with only local enhancement. Each forager is indicated by a SEARCH area (gray semi-circle), REACH (gray circle) and a movement trajectory (red to blue line). For illustration purposes, the resource items are shown as colored circles, and patches by a larger gray circles. Each patch can be assumed to be a distinct patch type, with unique resource types (different colours within a patch). (MP4 7390 kb)


**State variables:** Resources items are defined by a position, and a type which is characterized by quality *Q*_*r*_, and two parameters defining how difficult the resource type is to process (*H*_*r*_ and *S*_*r*_), or ‘task difficulty’. *H*_*r*_ defines the practice time (or experience) needed to develop half of the maximal skill for that resource type, and *S*_*r*_ defines the shape of the function of how skill increases with experience (see ‘Skill learning’ below). Patches are emergent from clumps of resource items in space, and have a type defined by a set of 5 resource types that only occur in patches of that type. Foragers are defined by a position and heading, a current action and a time to its completion, short-term memory about movement and foraging goals, and long-term memory about the rewards associated with resources and resource processing skill. Foragers can differ in their information about resources and skill levels, as well as in their propensity for learning as defined by parameters that can mutate (see Table 1).

**Processes and scheduling:** The implemented processes in our model are: (i) local decision making and movement of foragers; (ii) learning; (iii) life-history updating and demographics; and (iv) environmental updating.

Local decision making is governed by a decision-making algorithm which encodes sensing, decision making, movement, grouping and the updating of short-term memory. In simulations with grouping, foragers belong to a particular group, and follow behavior rules that ensure that groups move cohesively through the environment. All foragers are placed in a queue according to the time their action ends. The forager with the least time remaining is next to choose an action and is put back in the queue according to the time its new action ends. In this event-based setup, actions of foragers can overlap in time, and some foragers can complete multiple quick actions (e.g. move) while others are engaged in actions that take more time (e.g. searching for food).

The learning algorithms include representations of individual and social learning, and update long term memory about properties of resources that foragers interact with as a consequence of their decisions.

Life-history updating occurs at regular time intervals and includes: (i) metabolism or energy expenditure; (ii) digestion of consumed resources; (iii) deaths and (iv) births of foragers; and (v) splitting of groups. After a forager dies, a forager is selected from the remaining population to reproduce, thus maintaining a fixed population size. Foragers are selected to reproduce in relation to their energy levels, where a doubling in energy leads to an 8-fold increase in the probability to reproduce. Offspring inherit the parameter values of their parents with a chance of mutation (see Table 1). In simulations with grouping, groups grow due to births until they reach a maximum size, and then split randomly into two equally sized daughter groups. Groups shrink due to deaths and disappear when the last group member dies.

Environmental updating occurs at regular intervals and involves the ‘growth’ of all resource items at the beginning of each year and ‘environmental change’ that changes an existing resource types into an unknown (for foragers) new resource type. ‘Resource consumption’ occurs when foragers consume resources as determined by ‘local decision making’.

**Spatio-temporal scaling:** The environment is a continuous space of about 40 km ^2^, foragers take steps of a meter at a speed of 0.5 m/s, and patches are 20 meters in diameter (Fig. 1 a and Additional file 2). Foragers can observe resources up to 2 meters away, and can observe which resources their neighbors are interacting with at 20 meters (a best case scenario for social learning, Additional file 3). There are no constraints on observing group members for grouping purposes in order to ensure cohesive groups, but the spread of groups tends to be in the order of 5–40 meters. All movement occurs in continuous space and there are no constraints on direction.


Additional file 3: Video showing group foragers observing each other, which relevant for both stimulus enhancement and observational learning. Each forager is indicated by a SEARCH area (gray semi-circle), REACH (gray circle) and a movement trajectory (red to blue line). When a forager observes another forager the foragers are connected by an olive-green line. For illustration purposes, the resource items are shown as colored circles, and patches by a larger gray circles. Each patch can be assumed to be a distinct patch type, with unique resource types (different colours within a patch). (MP4 2370 kb)


The timescale is defined in terms of the foragers’ behavioral actions that vary in duration from about a few seconds to a minute. In the model a year is defined as 360 days, and a day is 12 hours or 720 minutes, where we focus on daylight time in a day. Thus foragers can complete many hundreds of behavioral actions in a day and learn from them. Energy expenditure (metabolism) occurs every minute. Digestion occurs every 100 minutes (DIGESTIONTIME). Foragers can live maximally for 20 years, but can die before that at any minute.

### Resources

In our default setting, resource items of 250 resource types are distributed in 24500 patches with 1200 items each. There are 50 patch types, and a patch type is characterized by the presence of five resources types that only occur in that patch type (as in trees with fruit, leaves, flowers etc). In order to generate variation across patches of a given type, each patch of a given type is defined by three resource types which are randomly selected from the five resource types that characterize that patch type. While these parameter values typically underestimate the diversity of natural environments, we strike a pragmatic balance between model complexity and simulation environments that are too simple, and where learning hardly plays a role [24]. We compare this ecological context with randomly distributed resources without patches, and pure patches where each patch type has only one resource type.

Resource items disappear when consumed by foragers, and are then unavailable for consumption. Resource ‘growth’ happens once a year, when all resource items that have been consumed by foragers reappear in the exact same position (for computational reasons) and with the same type. Environmental change occurs randomly at any minute with a given probability and changes a randomly selected resource type into another newly generated resource type which is unfamiliar to the foragers. For ease of interpretation we express this as a rate, namely how many resource types change per year (*EC*). All resource items of the disappearing type change into the new resource type. We vary environmental change *EC* across simulations to determine the effect of environmental change. We compare this kind of environmental change to one where resources do not disappear and change into new ones, but where resources remain familiar but change in quality.

The quality of a resource type *Q*_*r*_ is drawn from a random distribution with mean 0.1 and standard deviation of 0.1 (Fig. 1 b light gray), and all items of a given resource type have the same quality. Thus we generate variation in quality across resource types which enables the learning process to be studied as an optimization process. Quality defines the maximal reward that a forager can obtain from a resource type when it has sufficient experience with processing that resource type. Task difficulty is defined by *H*_*r*_, the practice time (or experience) needed to obtain half of the maximal reward of that resource type, and *S*_*r*_, which defines how the reward increases with experience (see ‘Skill learning’ below). *S*_*r*_ varies randomly between 1 and 4 (integer values only) and *H*_*r*_ is varied across simulations to determine an overall difficulty of learning in the environment.

### Local decision making

Foragers can choose between several local actions, namely, MOVE, SEARCH, MOVETOFOOD, EAT, MOVETOGROUP, OBSERVE and NOTHING, which are selected according to a decision-making algorithm (Fig. 1 b). In the algorithm, individuals start by checking if they are safe (CHECKSAFE), which implies having a sufficient number of neighbors (9) in SAFESPACE (17 meters). During CHECKSAFE, foragers can also observe group members within COPYSPACE (20 meters), and can monitor the resources with which those neighbors interact (Fig. 1 a). These observations are relevant for stimulus enhancement (SE) and observational learning (OL).

If not safe, foragers do MOVETOGROUP, which means that a forager moves towards the center of its group, calculated as the mean position of the other members of its group (Fig. 1 b, first line). Once safe, the forager then aligns its own heading with the average direction of other members of its group in ALIGNSPACE (20 meters). This attraction-alignment algorithm ensures that foragers stay together but travel in a relatively efficient manner through the environment.

If safe, foragers do OBSERVE (*τ*_*i*_ minutes) with probability *ω*_*i*_, which leads to observational learning (OL, see below; (Fig. 1 b, second line). Otherwise, with probability 1−*ω*_*i*_, foragers will select one of the remaining actions. If foragers are not HUNGRY (stomach content is at a maximum capacity of 20 resource items), foragers will do NOTHING (1 min; Fig. 1 b, third line). Stomach contents are reset to zero at DIGESTIONTIME.

If HUNGRY, and if they have already selected a resource item for consumption (FOODTARGET), foragers will EAT (1 min), or MOVETOFOOD if the item is beyond reach (0.9 meters) and EAT once the item is within reach (Fig. 1 b, fourth line). If foragers do not yet have a FOODTARGET but their last action was SEARCH, this means they did not find any resource items in view sufficiently attractive and then they will MOVE forward *δ*_*i*_ meters in the direction the foragers are facing (Fig. 1 b, fifth line). If they did not yet SEARCH, they will SEARCH (Fig. 1 b, sixth line). During SEARCH up to 20 resource items in view (2 meters) are assessed in sequence (Fig. 1 a, grey semi-circles). The 20 items are randomly selected from those in view. The search terminates as soon as an item is chosen for consumption, or when none of the items is chosen.

### Food choice algorithm

During SEARCH, a forager’s decision to EAT a given resource item is determined by its (i) exploration tendency *P*_*E*_ (see below), (ii) personal information about the rewards associated with that resource type (*a*_*ir*_), and (iii) whether the forager has been socially stimulated by seeing another forager eat that resource type *P*_*S*_ (see below). During evaluation of a resource item, these three factors come together to determine the probability *P*_*F*_ to choose to eat that item as follows:

1 $$ {\begin{aligned} { P_{F} =} P(r | a_{ir}, a_{ie}, \sigma_{i}, P_{E}, P_{S}) = min\left[ 1.0, \left(\frac{a_{ir}}{a_{ie}} \right)^{\sigma_{i}} + P_{E} + P_{S} \right] \end{aligned}}   $$

where *a*_*ir*_ is the reward forager *i* expects from resource type *r* (personal information based on reinforcement learning), *a*_*ie*_ is an assessment of the quality of resources that can be found in the environment (see below), and *σ*_*i*_ scales selectivity, i.e. how likely an individual selects when the expected reward *a*_*ir*_<*a*_*ie*_ (the expected quality of resources in the environment). Since associations are initially zero (*a*_*ir*_=0), unknown resource types can only be sampled via exploration (*P*_*E*_) or social stimulation (*P*_*S*_). For solitary foragers this means that the exploration rate *P*_*E*_ must be greater than zero. For grouping foragers, social stimulation *P*_*S*_ could in principle replace exploration *P*_*E*_ as the means to sample unknown resources. Once expected rewards *a*_*ir*_>0, $\left (\frac {a_{ir}}{a_{ie}} \right)^{\sigma _{i}}$ contributes to the probability of choosing a certain resource type, which is maximal when *a*_*ir*_>*a*_*ie*_ and less than one if *a*_*ir*_<*a*_*ie*_. If *a*_*ir*_>*a*_*ie*_, the forager is certain to choose the resource item, irrespective of *P*_*E*_ and *P*_*S*_. The impact of *P*_*E*_ and *P*_*S*_ is therefore greatest when resource are relatively unfamiliar (*a*_*ir*_<*a*_*ie*_).

Selectivity is adjusted relative to environmental conditions by adjusting the expected quality of the environment *a*_*ie*_ (Fig. 1 d, compare dark gray and black). When a forager’s stomach is not full at DIGESTIONTIME, the forager decreases its environmental expectation: *a* *ie*′=(1.0−*ϕ*_*i*_)*a*_*ie*_; otherwise the expectation is increased: *a* *ie*′=(1.0+*ϕ*_*i*_)*a*_*ie*_, where *ϕ*_*i*_ determines the rate with which the expected quality of the environment *a*_*ie*_ is changed. Each time the forager is too selective, it does not fill its stomach and reduces its selectivity, and vice versa. As a result, *a*_*ie*_ is tuned in order to optimise energy intake, within the constraints of the algorithm. Qualitatively, this selection algorithm can give rise to the optimal food choice rule [27] where only resources above a certain perceived quality are eaten and all others are ignored (zero-one rule). Note however that our algorithm works on perceived quality and not actual quality since the foragers are learning about resource quality and are not omniscient. Moreover, we let selectivity parameter *σ*_*i*_ evolve, so that while the zero-one rule is possible, it need not evolve and we don’t restrict the selection algorithm in this sense.

**Satiation aversion:** foragers develop temporary aversions after becoming satiated (stomach filled) with a given resource type. Satiation aversion causes foragers to completely ignore that resource type for one DIGESTION cycle (100 minutes) after which the aversion disappears. Satiation is common in foragers like primates that consume many secondary ‘toxic’ compounds [28], and/or require a balanced diet [29]. This model specification was added to ensure that foragers consume a diverse set of resource types [21], as is typical for primates and as was assumed in previous models [21, 24].

### Learning

In the absence of any social influences on learning, learning in our model is composed of (i) exploration, (ii) skill learning, and (iii) reinforcement learning about rewards associated with resources. All foragers start life without any knowledge about resources, and so do not have any expectation about energy rewards (*a*_*ir*_=0) nor any resource processing skill. To enable foragers to sample (partially) unfamiliar resource types, and hence to start learning, we implemented exploration. After processing resource items, foragers develop skill, which increases the rewards they can obtain from resources items of that type. After consuming resource items, foragers develop expectations about rewards via reinforcement, and can use those to decide what to eat. Note that for simplicity we do not include ‘forgetting’, and acquired skills and reward estimates are maintained indefinitely. We do not expect this to affect the results qualitatively.

**Exploration:** The probability that a forager explores an item of resource type *r* is:

2 $$ P_{E} = P(r | \varepsilon_{i}, c_{ir}) = \varepsilon_{i} (1 - c_{ir})   $$

*ε*_*i*_ is the exploration rate, and *c*_*ir*_ is the certainty with which forager *i* assesses the reward of resource type *r*. Certainty was included to ensure that foragers do not continue exploring when already highly familiar with resources. For completely unfamiliar resources *c*_*ir*_=0 and there is no certainty. However, when rewards from resource types no longer change, for instance because skill levels are high, certainty becomes high, and foragers end up with a low tendency to explore that resource type. Certainty *c*_*ir*_ is updated as follows:

3 $$ c_{ir}'=(1-\lambda_{i})c_{ir}+\lambda_{i}\left(1-min\left(1.0,\left|\frac{e_{ir}-a_{ir}}{e_{ir}}\right|\right)\right)   $$

where *e*_*ir*_ is the reward forager *i* obtains from resource *r*, and the same learning rate (*λ*_*i*_) and discrepancy between actual and expected rewards (*e*_*ir*_−*a*_*ir*_) are used as during updating of expected rewards (see Eq. 6).

**Skill learning:** A forager *i*’s skill *s*_*ir*_ for processing a specific resource *r* is a function of experience *t*_*ir*_ and ‘task difficulty’:

4 $$ s_{ir} = \frac{t_{ir}^{S_{r}}}{H_{r}^{S_{r}} + t_{ir}^{S_{r}}}   $$

which is 0 when experience *t*_*ir*_=0 and tends to 1 when *t*_*ir*_ becomes very large. Experience *t*_*ir*_ is the total time a forager *i* has spent processing a resource type *r* in its life, and increases each time the forager processes and consumes a resource item of type *r*.

Skill *s*_*ir*_ determines the reward *e*_*ir*_ forager *i* obtains from resource type *r* as a function of resource quality *Q*_*r*_:

5 $$ e_{ir} = Q_{r} s_{ir} + N(0, Z)   $$

where *N*(0,*Z*) represents environmental noise, where a value is drawn from a normal distribution with mean 0 and a standard deviation of *Z* (0.005). Resource types with high *H* (Fig. 1 c, dashed lines) take longer to learn, while resource types with high *S* have a shallow increment in rewards during initial learning (Fig. 1 c, gray lines). Note that for simplicity we assume that while for different foragers the same resource items can provide different energy, depletion from the environment and the number of items that can be eaten is the same. This can be interpreted as foragers consuming a certain amount of resource in a given amount of time irrespective of how well it is processed, but that energy obtained depends on processing. Moreover the item is then no longer available for other foragers.

**Reinforcement learning about expected rewards:** The rewards that foragers associate with each resource type *r* are updated via reinforcement as follows:

6 $$ a_{ir}' = a_{ir} + \lambda_{i}(e_{ir} - a_{ir})   $$

where association *a*_*ir*_ is the reward that forager *i* associates with resource type *r*, *e*_*ir*_ is the energy obtained from resource type *r*, and *λ*_*i*_ is the learning rate. This corresponds to a Rescorla-Wagner model [30] where all stimuli have the same salience. Associations are initially non-existent (i.e. zero), and the reward is obtained immediately after consumption of the resource leading to direct reinforcement.

### Social influences on learning

**Local enhancement (LE):** Arises spontaneously through grouping behaviour, since individuals are inclined to approach locations in which other members of their group are found, and thereafter to interact with resources in those regions. We therefore do not directly implement local enhancement, but it emerges spontaneously as soon as foragers move in groups [24]. The local enhancement that we consider is coarse grained, and does not direct individuals to particular resources, or to features of those resources.

For the two other social learning mechanisms, during CHECKSAFE a random ‘demonstrator’ is selected from any neighbors in COPYSPACE (see ‘Forager behavior’) that are processing and consuming a resource. The impact of the demonstrator depends on the social learning mechanism.

**Stimulus enhancement (SE):** In addition to selecting resources according to their expected reward and the tendency to explore a given resource type asocially, SE increases a forager’s probability to consume resource type *r* by:

7 $$ P_{S} = P(r | \gamma_{i}, d) = d \gamma_{i}   $$

where *γ*_*i*_ indicates the strength of SE, and *d*=1 if forager *i* observed a neighbor consuming resource *r* within the last 30 minutes and otherwise *d*=0. Only one resource type *r* is subject to SE at a time. SE does not directly affect expected rewards or skill.

**Observational learning (OL):** Occurs during the action OBSERVE at rate *ω*_*i*_ (see ‘Forager behavior’) and allows forager *i* to increase its processing skill for a specific resource type, in proportion to the time spent observing, where the change in experience *Δ* *t*_*ir*_ is:

8 $$ \Delta t_{ir} = max\left[K \frac{o_{ik}}{M}(t_{kr} - t_{ir}),0.0\right]   $$

where *K* scales the increase, determining how effective skill copying is, and *o*_*ik*_ is the effective time forager *i* observes neighbor *k*: *o*_*ik*_=*m* *i* *n*[*τ*_*i*_,*p*_*k*_], where *τ*_*i*_ is the maximum time forager *i* decides to spend observing its neighbor, and *p*_*k*_ is the time left for neighbor *k* to complete its present action. Greater observation time leads to greater skill acquisition, where maximal observation time is the maximal time it takes to process and consume a resource (*M*). The increase in the skill level is bound to the skill level of the observed individual, and there is no skill gain if the skill level of the observed individual is lower than, or equal to, the forager’s own skill level. A forager does not know in advance whether a ‘demonstrator’ is highly skilled or not. Observation does not provide information about rewards.

### Energy budget, population turn-over and selection

The energy budget is determined by (i) energy gain due rewards from food intake which depends on learning at every DIGESTIONTIME, (ii) a per minute energy metabolism cost (METABOLISM, see Section 1 in Additional file 1), and (iii) an energy costs of 5000 for a reproduction event, which represents a substantial part of total energy. Energy accumulates if energy intake from food exceeds metabolism and reproduction costs. A limited stomach capacity and digestion intervals were added to the model to ensure selective foraging, as is typical for primates and as was assumed in previous models [21, 24]. In addition, an explicit metabolism cost, ensures that there is a viability constraint in the model, where foragers must gain enough energy from food otherwise they die.

Foragers die of old age (at 20 years), stochastically determined deaths, or starvation. Births occur as a function of energy reserves each time a forager dies, keeping the population constant at size *N* (100), where probability that forager *i* reproduces is:

9 $$ P_{R} = P(i | N) = \frac{h_{i}^{W}}{\sum_{j=1}^{N} h_{j}^{W}}  $$

where *h*_*i*_ is an individuals energy level, *N* is the population size, and *W* (=3) scales the strength of the selection function.

The learning and foraging parameters *δ*_*i*_, *ϕ*_*i*_, *σ*_*i*_, *ε*_*i*_, *λ*_*i*_, *γ*_*i*_, *ω*_*i*_, *τ*_*i*_, are specific to forager *i*. Parameter combinations that lead to greater energy levels lead to faster rates of reproduction. An offspring inherits its parent’s parameters, with a chance of mutation (0.05). In case of mutation, a new parameter value is drawn from a normal distribution centered on the parent’s parameter value, and with a standard deviation that is one fifth of the maximum value of the parameter (see Table 1). Thus parameters can vary between individuals and can evolve over time via inheritance to offspring, mutation and natural selection. The mutation rate was selected operationally such that parameters evolve consistently within a reasonable time frame.

Foragers are born in their parent’s group. There is no migration between groups. The population is inviable if the average energy level does not rise above the minimum energy needed to give birth.

### Emergent dynamics

Since we only define local sensing and behavioral actions of foragers, the development of a forager’s repertoire emerges from its interaction with the environment over time. This environment includes the resources and their distribution, which affects the temporal autocorrelations in encounters with resources. The movement of foragers is characterized by inter-patch travel where no resource items are found, and intra-patch search, assessment and consumption of resource items. Within each patch, a forager has access to the resource types that are present in that patch. Over their lifetime, foragers encounter all patch types and all the resource types they contain, many times, thus there is ample opportunity to consume all resource types repeatedly. On reaching a patch, a forager’s experience with those resource types will depend on previous encounters with those resource types, and if it consumed those resources in the previous digestion cycle it could be satiated with respect to those resource types.

The dynamics of foraging are characterized by learning and food choice [21, 24]. Foragers move through the environment and when they encounter resource items, the food choice algorithm determines whether any are consumed (Eq. 1). Foragers start out exploring various unknown resources (via *P*_*E*_ and/or *P*_*S*_), and as they gain experience about rewards, personal information tends to become more dominant in their food choices. Personal experience is updated after consumption events and includes *a*_*ir*_, the assessment of rewards (Eq. 6) and the increment of skill (Eq. 4) which in turn increases the reward obtained (Eq. 5). Due to consumption of many resources, the expectation of the environment *a*_*ie*_ will increase, increasing the fraction of resources for which *a*_*ie*_ is greater than an expected reward *a*_*ir*_. This increases selectivity towards resources with high expected reward *a*_*ir*_, and can lead to reduced food intake (i.e. a forager’s stomach is no longer full at digestion). At this point the expectation of the environment *a*_*ie*_ decreases again.

Thus the forager’s expectation of the environment *a*_*ie*_ tends to equilibrate on a value in relation to values of *a*_*ir*_, such that the intake of resource items is close to the maximum of 20. This ensures that the forager is eating selectively but still eating close to the maximal number of resource items within each digestion cycle (DIGESTONTIME). The ratio of *a*_*ir*_ to *a*_*ie*_ is therefore similar across simulation types, irrespective of how fast *a*_*ir*_ increases due to differences in skill development time.

The combination of (i) food choice biased to resource types with high expected reward *a*_*ir*_ (selective foraging), and (ii) learning via updating of *a*_*ir*_ and experience *t*_*ir*_, generates a positive feedback. This positive feedback generates a familiarity bias and a development process that is contingent on stochastic initial conditions, leading to idiosyncratic learning histories and somewhat arbitrary variation between foragers in their knowledge of the environment. Therefore, while learning is biased towards high quality resources, due to an intrinsic familiarity bias in the process, learning could get ‘stuck’ on a self-stabilizing repertoire as soon as this repertoire fulfills the intake needs of the forager [24]. Previous work has shown that this familiarity bias becomes strong in environments with pure patches, and when foragers do not become satiated after eating a lot of a given food type [21, 24]. We therefore focus on patches with several resources and satiation as a default case, so that the familiarity bias is not unreasonably strong such that foragers only end up consuming a few resource types.

The familiarity bias implies that foragers have greater experience *t*_*ir*_ for some resources than others, and also a more accurate assessment *a*_*ir*_ of rewards *e*_*ir*_. Since learning rate *λ*_*i*_ typically evolves to high values [26], an expected reward *a*_*ir*_ is generally an accurate estimate of the actual reward *e*_*ir*_. The main cause for differences in familiarity is therefore differences in processing experience *t*_*ir*_ and these determine differences between reward *e*_*ir*_ and expected reward *a*_*ir*_. As a result, the impact of social influences on learning therefore concern (i) biases on choosing resource types, which indirectly affect experience *t*_*ir*_ in the case of LE and SE, and (ii) direct gains in experience *t*_*ir*_ in the case of OL.

In groups, the actions of neighbors and group-level dynamics can have indirect and direct influences on food choices and learning [24]. Due to the need to stay in a group (imposed in the model), there is a strong ‘consensus’ or ‘conformity’ effect, where the decision of neighbors to stop or not stop in a patch can affect the feeding opportunities of foragers and hence their learning trajectories. This social influence on learning due to grouping, which we refer to as LE, is an emergent process in our model. This process occurs in patchy environments, because grouping causes foragers to share the same foraging opportunities at the same time. As a result, foragers in groups share learning histories and develop similar behavioral repertoires [24]. Moreover, the direct observation of neighbors and its effects, depends on what neighbors have decided to eat, or depends on copying opportunities [22]. In turn, the effect of a social stimulus will depend on what an observer already knows, and whether it can find the resource type of interest. If a forager would already choose a resource item on its own accord (*a*_*ir*_>*a*_*ie*_) then the social stimulus *P*_*S*_ would not matter and the social influence would be redundant. Moreover, *P*_*S*_ can increase the rate of food intake and feedback on selectivity via the updating of the expectation of the environment *a*_*ie*_.

When naive foragers are introduced due to population turnover, and these follow the group, they end up spending time in patches that are already preferred by experienced foragers. As a result, their development is biased towards resources that the group already consumes. Since familiarity and preference are self-reinforcing (also emergent in the model) the young foragers could end up developing the same preference biases and so can end up inheriting their group’s behavioral repertoire [21]. If behavioral repertoires are unique to a given group and persist across generations, then this can be seen as traditional differences between groups or cultural variation.

In addition to developing more or less the same behavioral repertoire as their group, young foragers could also become more selective than older foragers, resulting in their rejection of the lower quality resources in the group repertoire [21]. This is possible because young foragers experience a different ‘frame of reference’ with respect to their repertoire development than older ones have, where young foragers can select a subset within the repertoire of their group. In addition, young foragers could add resource types to their repertoire that are novel for the group since initially they do not have a familiarity bias. However, these resource types will only be selected if they are considered to be sufficiently rewarding relative to others in the repertoire. Hence this process tends to lead to the inclusion of relatively high quality novel resource types. In sum, both the rejection of low quality resource types and the inclusion of high quality ones, can generate a process whereby the repertoire quality in the group improves over generations beyond the lifespan of a single forager. This can be seen as a cumulative cultural process [21]. Note that this process will mainly occur in the early stages when a group explores a new environment. After a while the cumulative process levels off, and new generations no longer become more selective than previous generations.

### Simulations and analysis

In a previous study we used the same model to establish the evolutionary attractors in different environmental conditions, and determine the payoffs and information production associated with different social learning mechanisms [26]. These parameters define foraging and different (social) learning mechanisms in the foragers. The evolved learning parameters are exploration (*ε*_*i*_), stimulus enhancement (*γ*_*i*_) and observational learning (*ω*_*i*_ and *τ*_*i*_). The evolved foraging parameters (*δ*_*i*_, *σ*_*i*_, *ϕ*_*i*_, *λ*_*i*_) ensure that the foraging and reinforcement learning parameters are not arbitrarily defined, but have co-evolved with the main parameters of interest. Here we studied whether and how these evolved parameters lead to traditional differences between groups (cultural variation) and cumulative cultural change in energy intake.

In our analysis we focused on questions that arose from expectations based on previous research (see Introduction). To address these questions we used non-evolutionary simulations initialized with evolved parameters to measure diet repertoire statistics in more detail. To study the effect of protracted learning, we varied the task difficulty of resources (*H*_*r*_).

We consider traditions to be between-group differences that are inherited over time due to social learning. To quantify to which extent between-group differences are inherited we combined (1) a measure of within-group repertoire similarity across time, and (2) a measure of between-group differences in repertoires at a given point in time. Within-group similarity across time on its own is insufficient for identifying inheritance, since next to social learning, within-group similarity can also be generated if all individuals converge on feeding on the same high quality resources due to repertoire optimization. Thus we needed to establish that the group-level repertoires that were maintained over time were distinct from those of other groups, hence ruling out population-level convergence due to factors such as repertoire optimization.

To do so we calculated the difference between within-group similarity over time and between-group similarity at one specific point in time [21]. We calculated within-group similarity over time as the overlap in repertoires at year 120 and 100. This 20 year period ensured there is no overlap in foragers at the two time points. For between-group similarity we calculated overlap between a group and other groups in an independent simulation with the exact same environment. In this way we controlled for relatedness between groups, and competition between groups, which increase and decrease between-group similarity respectively. We calculated average repertoire similarity between groups *k* and *l* as:

10 $$ \bar{O}_{k,l} = \frac{1}{G_{k} G_{l}}\sum_{i=1}^{i=G_{k}} \sum_{j=1; j!=i}^{j=G_{l}} \frac{\vec{d_{i}}\bullet\vec{d_{j}}}{|\vec{d_{i}}|.|\vec{d_{j}}|}  $$

where $\vec {d_{i}}$ is the vector of number of items eaten per resource type (behavioral repertoire) of forager *i*, $\vec {d_{i}}\bullet \vec {d_{j}}$ is the dot product of the behavioral repertoires of foragers *i*, *j*, $|\vec {d_{i}}| = \sqrt (\sum _{r=1}^{r=R} |d_{i,r}|^{2})$ is the length of vector $\vec {d_{i}}$, *R* is the number of resources types, and *G*_*k*_ is the number of foragers in group *k*. This function returns a value of 1 if the group-level repertoire is identical in both groups (i.e. either for the same group at different point in time or between two groups at the same point in time), and it returns a value of 0 if there is no overlap in repertoires (i.e. the none of the resources in one repertoire exist in the other repertoire).

For cumulative cultural change we focused on energy intake over time. We used a conservative approach to focus on whether cultural processes enable phenotypes that are beyond what foragers can achieve within a single lifetime. We therefore considered the difference between (1) year 20, which represents the maximum that individuals can achieve within their lifetime, and (2) year 120, the end of our simulation by which time the cumulative process had levelled off. The change was expressed as a proportion of the measure at year 20, where total energy intake is calculated as follows: (i) total energy intake $= \sum \limits _{r=1}^{r=R} d_{ir} e_{ir}$, where *d*_*ir*_ is the total number of items of resource type *r* that were consumed by forager *i*, and *e*_*ir*_ is the per item reward obtained. We repeated this analysis on other repertoire measures in order to analyze whether skill, repertoire quality and repertoire diversity also change cumulatively: (ii) repertoire quality $= \sum \limits _{r=1}^{r=R} {z_{ir}} Q_{r}$; (iii) repertoire diversity $= \sum \limits _{r=1}^{r=R} - {z_{ir}} \log {z_{ir}}$; (iv) average skill $= \sum \limits _{r=1}^{r=R} {z_{ir}} s_{ir}$, where *z*_*ir*_ is the proportion of resource *r* in individual *i*’s diet, *Q*_*r*_ is the quality of resource type *r*, and *s*_*ir*_ is the skill forager *i* has for resource type *r*. Note that for repertoire diversity we only considered resource types that had been consumed (i.e. *z*_*ir*_>0). In contrast to measuring traditional differences, we used different random seeds for the environment in each simulation so as to not repeat the exact same pattern of environmental change.

As a default we considered patchy environments with multiple resource types in each patch (mixed patches) and with a low but reasonable rate of environmental change [25]: a random 5 resource types per year were replaced with a new kind of resource type with a randomly assigned resource quality *Q*_*r*_. We did not vary parameters that defined life-history characteristics and spatio-temporal scaling as this is beyond the scope of study. In sum, while the analysis contained a large number of parameters, the vast majority of these provide a realistic simulation context, and the parameter space for the remaining few was explored within realistic bounds [26].

## Results

### Do all the SLMs tested generate traditional differences?

**Fig. 2:**
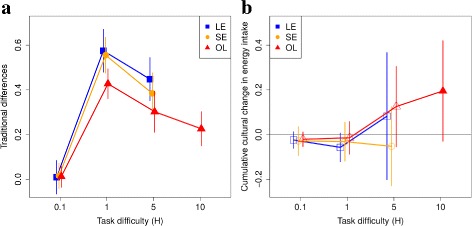
Traditional differences **a** and cumulative cultural increase in energy intake **b** as a function of SLMs and task difficulty (*H*) for EC=5. Blue = LE, Orange = SE, and Red = OL. Solid symbols represent those cases where we find a statistically significant increase in energy intake (Wilcoxon signed rank test with continuity correction and a Bonferroni corrected *α* level of 0.0125 to maintain a familywise error rate of 0.05). LE and SE are inviable at H10 and are excluded. Each data point is the mean of 10 simulations, and whiskers show standard deviation. The calculation of traditional differences and cumulative cultural change is explained in the ’Simulations and analysis’ section

We find that all SLMs can generate traditional differences under a wide range of environmental conditions. In Fig. 2 a we show average levels of traditional differences between groups. We show statistically significant increases in traditional differences using solid symbols (Fig. 2, Wilcoxon signed rank test with continuity correction and a Bonferroni corrected *α* level of 0.0125 to maintain a familywise error rate of 0.05). Statistically significant traditional differences are generated for all *H* conditions, but are non-negligible for *H*>0.1 (Fig. 2 a). At *H*=0.1 we do obtain statistically significant results since the distribution is skewed to be above zero, but the magnitude of traditional differences is very small.

### Do all the SLMs tested generate cumulative cultural change?

In contrast to traditional differences, cumulative cultural change is restricted to OL for a narrow range of environmental conditions. In Fig. 2 b we show average levels of cumulative cultural increases in energy intake. Statistically significant cumulative cultural change is only generated for OL and only for *H*=10 (Fig. 2 b, solid triangles, Wilcoxon signed rank test with continuity correction and a Bonferroni corrected *α* level of 0.0125 to maintain a familywise error rate of 0.05). Note that foragers in groups with LE or SE are not viable at *H*=10 [26] and are excluded.

### Does task difficulty enhance cultural phenomena?

Our results confirm that learning needs to be sufficiently protracted to generate traditions and cumulative cultural change. In Fig. 2 we can observe that task difficulty must be sufficiently high, (i.e. learning must be sufficiently protracted, before these cultural phenomena are generated, *H*>0.1). At *H*=0.1 learning is very easy and all foragers are effectively all knowing and all the groups are the same and there are no traditional differences. Once learning is sufficiently difficult (*H*>0.1) traditional differences can arise. However, the specific effects of different task difficulties varies between traditions and cumulative cultural increase, where the latter requires very high task difficulty before is detectable. Moreover, increasing task difficulty beyond *H*=1 does not necessarily lead to greater traditional differences, and after *H*=1 traditional differences actually level off or even decline. This occurs due to population-wide convergence on resource types that are easy to learn which becomes increasingly pronounced as task difficulty increases (see Section 2 in Additional file 1 for more details).

### Do SE and OL enhance traditional differences and cumulative culture?

In contrast to our expectation, we did not observe that SE and OL enhance traditional differences compared to LE (Fig. 2 a). In fact the greatest traditional differences are found for LE. We also do not find that LE or SE generate cumulative cultural change (Fig. 2 b, squares and circles).

**Fig. 3:**
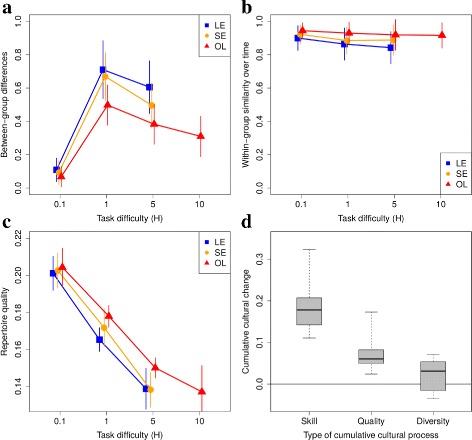
Between-group differences (1 - between-group similarity) (**a**), within-group similarity over time (**b**), repertoire quality **c** for different task difficulty (*H*), and different kinds of cumulative process for OL at *H*=10 (**d**). For **a**–**c**: *Blue* = LE, *Orange* = SE, and *Red* = OL. LE and SE are inviable at H10 and are excluded. Each data point is the mean of 10 simulations, and whiskers show standard deviation. For **d**: Boxplots show minimum, 1st quartile, median, 3rd quartile, and maximum, where data comes from 10 simulations. The calculation of repertoire statistics and cumulative cultural change is explained in the ’Simulations and analysis’ section

The reason that between-group differences are greatest for LE is that repertoire optimization is lowest for LE (Fig. 3 c, compare blue to orange and red). In SE and especially OL, greater repertoire optimization [26] leads to convergence in repertoires between groups, diminishing between-group differences. For between-group differences, we find nearly the exact same pattern as for traditional differences (compare Fig. 3 a with Fig. 2 a). For within-group similarity over time, we find that similarity is high overall (Fig. 3 b), but lowest for LE (blue), and greatest for OL (red). Given that we calculate traditional differences based on (1) between-group differences (1 - between-group similarity) and (2) within-group similarity across time, between-group differences are the main determinant of traditional differences in our results. Thus, even though LE exhibits the lowest within-group similarity, due to large between group differences, LE exhibits the greatest traditional differences.

### Do cumulative cultural increases in skill level and repertoire quality contribute to energy intake?

As expected, we find that when OL generates a cumulative cultural increase in energy levels, this is accompanied by a cumulative cultural increase in skill level (Fig. 3 d). In Fig. 3 d we can observe that OL at *H*=10 leads to a large increase in skill level. We find that next to increases in skill, OL at *H*=10 also leads to cumulative cultural increases in repertoire quality (Fig. 3 d). Thus the increases in energy intake result from both increases in skill level and repertoire quality.

In previous research we found that decreases in repertoire diversity could lead to increases in skill level, because a narrower repertoire enables greater skill development per resource type [26]. In Fig. 3 d we show that there is no decline in per capita repertoire diversity as skill levels increase (blue). This indicates that the increase in skill levels occurs via direct effects, where OL enables foragers to shortcut the developmental process by using the experience of other foragers. As a result they can achieve even greater levels of skill than the previous generations. Moreover, given that there is no decrease in per capita repertoire diversity (Fig. 3 d), the increase in repertoire quality implies a replacement, or even addition of high quality resources to the repertoire. We therefore see an increase in skill levels while repertoire diversity is maintained, which means that the overall level and quality of knowledge increases.

## Discussion and conclusions

Our results inform the debate over the cognitive requirements of culture. The findings are consistent with the idea that cognitively demanding SLMs are necessary for the generation of cumulative cultural change, but imply that traditions can result from simple SLMs. LE and SE can generate traditional differences between groups even though these basic SLMs do not affect skill learning directly. Our results support the idea that animal cultures will be widespread, but cumulative cultural change might be rare.

Overall our results support previous theory in the context of ‘learning what to eat’ [21] that predicts LE can suffice to generate traditional differences between groups in patchy environments. Here we demonstrate that this result also holds in the context of skill learning with variable group sizes, stochastic demographics and evolving parameters. These findings lend support to the idea that traditional differences between groups, even with respect to skill learning, do not rely on cognitively demanding forms of social learning [21, 23].

In contrast to previous ‘diet learning’ simulation results [21, 22] we found that LE and SE are not sufficient for generating cumulative cultural increases in energy intake. Instead, cumulative cultural change is limited to environments with very high task difficulty (*H*=10) and when foragers are capable of OL. Thus it is possible that previous results [21] are not robust to the change in learning context, and/or to one of the other assumptions that we changed in our model: stochastic population dynamics with variable group sizes, evolved parameters and environmental change. This will have to be determined by further model studies that revisit the ’diet learning’ context and investigate whether stochastic population dynamics with variable group sizes, evolved parameters and environmental change alter the previously found results. For now, we conclude that finding cumulative cultural increases in energy intake is likely to be context dependent. In particular, our results here support the idea that cumulative cultural change is promoted by cognitively demanding forms of social learning [4, 13].

In support of previous findings, we find that protracted learning is important for both cultural phenomena [21, 24]. On the one hand, protracted learning tends to limit the optimization of repertoires allowing for arbitrary variation between groups and hence traditional differences. On the other hand, the within-lifetime limitation of optimization of repertoires make cumulative cultural processes possible. In previous work we also found that protracted learning is important for the costs and benefits associated with particular SLMs [26]. Thus multi-scale models with protracted learning allow us to study the adaptive and cultural impact of particular SLMs rather than assume them. In this way multi-scale models can be used to evaluate the assumptions we make about social learning in various top-down verbal and formal models.

### Implications for the evolution of animal culture

Previously we found that LE did not increase energy intake relative to solitary foraging [26], and concluded that grouping would probably evolve for other reasons, for example as an anti-predation strategy [31]. If so, then our model here, like a previous model [21], predicts that traditional differences would evolve as a side-effect of grouping without any special cognitive adaptation besides those needed for living in groups.

Relative to this baseline of traditional differences as an evolutionary byproduct, we showed in previous work that SE and OL can readily evolve because they enhance the level of foraging efficiency [26]. Here we show that the evolution of such increased optimization need not generate greater traditional differences between groups, but could instead reduce them (Fig. 3 a). It is intuitive to assume that more accurate SLMs will increase within-group similarity (or conformity) and hence increase differences between groups [32]. However, this overlooks the effect of SLMs on enhancing repertoire optimization [26]. If all groups are able to correctly identify and eat the highest quality resources then behavioral repertoires will become similar [21], because the highest quality resources are always a limiting subset (Fig. 1 d). Despite this possible convergence between groups, we find that even when learning parameters evolve, optimization can still be sufficiently limited to allow for traditional differences.

While our findings support the idea that traditions should be widespread in foragers in cohesive groups living in patchy environments, for cumulative cultural change we expect a large context dependency. Previous theoretical work on diet learning showed that cumulative culture could arise as a side-effect of grouping and therefore might commonly occur in animal societies [21]. Our results here suggest that in the context of skill learning, cumulative cultural increases in energy intake may only arise for OL and only in environments with high task difficulty. The latter supports the idea that cumulative cultural processes may occur predominantly in species with cognitively demanding forms of social learning [4]. In particular, since SE and LE are inviable in environments with high task difficulty, our results suggest that OL would need to evolve before niches with high task difficulty could be invaded, and only thereafter would cumulative cultural increases in energy intake evolve. Previously we have argued that through this process, the evolution of cognitively demanding forms of social learning could open up novel niches [26]. Further modelling work is needed to confirm these expectations.

Our measure of cumulative cultural change is very general and does not necessarily imply (i) the generation of behavioral complexity via the invention of novel behavioral combinations, or levels technological of complexity, nor (ii) open-ended change [4, 13, 33, 34]. In our model, the latter cannot arise because novel behavioural opportunities cannot be generated, and the cumulative cultural process is restricted to the opportunities that are available in the environment, and is ‘bounded’. Thus, the complexity of behavior remains limited in the sense that any single behavior could be invented within the lifetime of an individual [4]. However, this behavior-level view contrasts with our repertoire-level perspective, where we consider culture cumulative if foragers exhibit a repertoire quality and overall skill level that they could not achieve within a lifetime of asocial learning. Thus while each single behavior could in principle be discovered by any forager, the level of repertoire optimization, or total ‘ecological knowledge’, cannot. In future, this ‘ecological knowledge’ perspective could be extended to spatial knowledge, in order to establish a more complete perspective on the scope of cumulative culture next to diet learning [21] and skill learning (present study) in group foragers.

Bounded contexts appear reasonable for considering cultural phenomena in many primate species [4] and the kind of bounded cumulative culture observed here provides a putative evolutionary precursor to more open-ended forms of cumulative culture. However, our results suggest that precisely because the cumulative process is expressed at a repertoire level and bounded, detecting existing cumulative cultural processes empirically may be very difficult. We would expect a bounded cumulative cultural process to operate for some time, but then level off. Thus when observing primates in the wild, researchers may well be measuring the outcome of a cumulative cultural process, where the phenotypes observed cannot be achieved within a single lifetime, even though changes in time may not be detectable. Moreover, quantifying the difficulty of acquiring a particular repertoire and detecting social influences is extremely difficult [35], which could help to explain the lack of empirical evidence for such cumulative processes. Studying the reintroduction of animal species to the wild may be a promising setting in which to study the possibility of cumulative cultural change in animals across generations. The difficulty of successful re-introductions to the wild, especially those in great apes [36], could be an indication of a dependence on cumulative culture.

If ecologically-bounded contexts are an evolutionary precursor to more open-ended forms of cumulative culture, then how can we use this to understand the transition between the two? At present many key variables have been proposed to explain this transition including, cognitive abilities for high fidelity copying [4, 13, 34], large population sizes [37, 38] and high rates of socialization and division of labour [33]. What is lacking at present is a framework that explains how these factors originate and co-evolve. Extensions to the multi-scale simulation model presented here could help to address this question. In this sense our model represents a tangible ecologically-bounded baseline in which researchers could study how ecological bounds could be relaxed. In particular, we expect that niche construction processes [39, 40] will be critical in relaxing the bounds found in our model, because these appear to be needed for generating feedbacks between cultural inheritance and opportunities for cultural innovation. In this way, cultural processes can start to define their own possibilities for change.

## Additional files

Additional file 1 The third file is Additional_file3.pdf in PDF format which can be viewed in any PDF-viewer such as Acrobat Reader. This file includes additional detail about the main simulation model and the modeling methodology and additional analysis. (PDF 243 kb)

## Abbreviations

LE: Local enhancement
OL: Observational learning
SE: Stimulus enhancement
SLM: Social learning mechanism

## References

[1] Perry S, Manson JH. Traditions in monkeys. Evol Anthropol. 2003; 12:71–81. doi:http://dx.doi.org/10.1002/evan.10105.

[2] Danchin E, Giraldeau LA, Valone TJ, Wagner RH (2004). Public information: from nosy neighbors to cultural evolution. Science.

[3] Tomasello M, Kruger AC, Ratner HH (1993). Cultural learning. Behav Brain Sci.

[4] Tennie C, Call J, Tomasello M (2009). Ratcheting up the Ratchet: On the Evolution of Cumulative Culture. Proc R Soc Biol Sci Ser B.

[5] Richerson PJ, Boyd R (2005). Not by Genes Alone. How Culture Transformed Human Evolution.

[6] Helfman GS, Schultz ET (1984). Social Transmission of Behavioural Traditions in a Coral Reef Fish. Anim Behav.

[7] Laland KN, Williams K (1997). Shoaling generates social learning of foraging information in guppies. Anim Behav.

[8] Panger MA, Perry S, Rose L, Gros-Louis J, Vogel E, Mackinnon KC, Baker M (2002). Cross-site differences in foraging behavior of white-faced capuchins (*Cebus capucinus*). Am J Phys Anthropol.

[9] Whiten A, Goodall J, McGrew WC, Nishida T, Reynolds V, Sugiyama Y, Tutin CEG, Wrangham RW, Boesch C (1999). Cultures in Chimpanzees. Nature.

[10] van Schaik CP, Ancrenaz M, Borgen G, Galdikas B, Knott CD, Singleton I, Suzuki A, Utami SS, Merill M (2003). Orangutan Cultures and the Evolution of Material Culture. Science.

[11] Krützen M, Willems EP, van Schaik CP. Culture and geographic variation in orangutan behavior. Curr Biol. 2011; 21(21):1808–12. doi:http://dx.doi.org/10.1016/j.cub.2011.09.017. 10.1016/j.cub.2011.09.017 22018539

[12] Rendall L, Whitehead H (2001). Culture in whales and dolphins. Behav Brain Sci.

[13] Dean LG, Vale GL, Laland KN, Flynn E, Kendal RL. Human cumulative culture: a comparative perspective. Biol Rev Camb Philos Soc. 2014; 89(2):284–301. doi:http://dx.doi.org/10.1111/brv.12053. 10.1111/brv.12053 24033987

[14] Heyes CM (1993). Imitation, culture and cognition. Anim Behav.

[15] Hoppitt W, Laland KN (2013). Social Learning: An Introduction to Mechanisms, Methods, and Models.

[16] Whiten A, Horner V, de Waal FBM. Conformity to cultural norms of tool use in chimpanzees. Nature. 2005; 437(7059):737–40. doi:http://dx.doi.org/10.1038/nature04047. 10.1038/nature04047 16113685

[17] Whiten A, Spiteri A, Horner V, Bonnie KE, Lambeth SP, Schapiro SJ, de Waal FBM. Transmission of multiple traditions within and between chimpanzee groups. Curr Biol. 2007; 17(12):1038–43. doi:http://dx.doi.org/10.1016/j.cub.2007.05.031. 10.1016/j.cub.2007.05.031 17555968

[18] Dindo M, Thierry B, Whiten A (2008). Social diffusion of novel foraging methods in brown capuchin monkeys (Cebus apella). Proc R Soc B.

[19] Dindo M, Whiten A, De Waal FBM (2009). In-group conformity sustains different foraging traditions in capuchin monkeys (Cebus apella). PloS One.

[20] Claidière N, Sperber D. Imitation explains the propagation, not the stability of animal culture. Proc R Soc B Biol Sci. 2010; 277(1681):651–9. doi:http://dx.doi.org/10.1098/rspb.2009.1615. 10.1098/rspb.2009.1615 PMC2842690 19889707

[21] van der Post DJ, Hogeweg P (2008). Diet traditions and cumulative cultural processes as side-effects of grouping. Anim Behav.

[22] van der Post DJ, Ursem B, Hogeweg P (2009). Resource distributions affect social learning on multiple timescales. Behav Ecol Sociobiol.

[23] Franz M, Matthews LJ (2010). Social enhancement can create adaptive, arbitrary and maladaptive cultural traditions. Proc R Soc Biol Sci Ser B.

[24] van der Post DJ, Hogeweg P (2006). Resource distributions and diet development by trial-and-error learning. Behav Ecol Sociobiol.

[25] van der Post DJ, Hogeweg P (2009). Cultural inheritance and diversification of diet in variable environments. Anim Behav.

[26] van der Post DJ, Franz M, Laland KN (2016). Skill learning and the evolution of social learning mechanisms. BMC Evol Biol.

[27] Charnov EL (1976). Optimal Foraging: Attack Strategy of a Mantid. Am Nat.

[28] Altmann SA (1998). Foraging for Survival.

[29] Provenza FD (1996). Acquired aversions as the basis for varied diets of ruminants foraging on rangelands. J Anim Sci.

[30] Rescorla RA, Wagner AR. A theory of Pavlovian conditioning: Variations on the effectiveness of reinforcement and non-reinforcement In: Black AH, Prokasy WF, editors. Classical Conditioning II: Current Research and Theory. New York: Appleton-Century-Crofts: 1972. p. 64–99. http://www.bibsonomy.org/bibtex/2686a54a423e9ce5d48c77222bc5a2722/josephausterwei.

[31] Krause J, Ruxton GD (2002). Living in Groups.

[32] Henrich J, Boyd R (1998). The evolution of conformist transmission and the emergence of between-group differences. Evol Human Behav.

[33] Pradhan GR, Tennie C, van Schaik CP. Social organization and the evolution of cumulative technology in apes and hominins. J Hum Evol. 2012; 63(1):180–90. doi:http://dx.doi.org/10.1016/j.jhevol.2012.04.008. 10.1016/j.jhevol.2012.04.008 22658335

[34] Lewis HM, Laland KN. Transmission fidelity is the key to the build-up of cumulative culture. Philos Trans R Soc Lond B Biol Sci. 2012; 367(1599):2171–80. doi:http://dx.doi.org/10.1098/rstb.2012.0119. 10.1098/rstb.2012.0119 PMC3385684 22734060

[35] Chapman CA, Fedigan LM (1990). Dietary differences between neighboring *Cebus capucinus* groups: local traditions, food availability or responses to food profitability?. Folia Primatol.

[36] Beck BB. Chimpanzee orphans: sanctuaries, reintroduction and cognition In: Lonsdorf EV, Ross SR, Matsuzawa T, editors. Chicago: University of Chicago Press: 2010.

[37] Diamond J (1978). The Tasmanians: the longest isolation, the simplest technology. Nature.

[38] Henrich J (2004). Demography and cultural evolution: how adaptive cultural processes can produce maladaptive losses: the Tasmanian case. Am Antiquity.

[39] Laland KN, Odling-Smee J, Feldman MW (2000). Niche construction, biological evolution, and cultural change. Behav Brain Sci.

[40] Laland KN, Olding-Smee J, Feldman MW (2001). Cultural niche construction and human evolution. J Evol Biol.

