# Supplementary material for: The evolution of social learning mechanisms and cultural phenomena in group foragers
Source: BMC Evol Biol. 2017 Feb 10;17:49. doi: 10.1186/s12862-017-0889-z (PMC5303232; doi:10.1186/s12862-017-0889-z)
Supplement: Additional file 1 — The third file is Additional_file3.pdf in PDF format which can be viewed in any PDF-viewer such as Acrobat Reader. This file includes additional detail about the main simulation model and the modeling methodology and additional analysis. (PDF 243 kb) [file 12862_2017_889_MOESM1_ESM.pdf]

Supporting information for "The evolution of social learning mechanisms and cultural phenomena in group foragers"

Daniel J van der Post[1*], Mathias Franz[2], Kevin Laland[1]
1 Center for Social Learning and Cognitive Evolution, St Andrews University, St Andrews, UK
2 Leibniz Institute for Zoo and Wildlife Research, Berlin, Germany
∗ E-mail: d.j.vanderpost@gmail.com

Contents

1 Detailed model description 2
1.1 Purpose . . . 2
1.2 State variables and scales . . . 2
1.3 Process overview and scheduling . . . 3
1.4 Design concepts . . . 3
1.5 Specification choices of scaling context . . . 5
1.6 Initialization . . . 6
1.7 Input . . . 6
1.8 Submodels . . . 6
1.8.1 Forager continuous updates and decision making algorithm . . . 6
1.8.2 Behavior actions of foragers . . . 7
1.8.3 Forager discrete updates . . . 9
1.8.4 Resources . . . 9
1.9 Comparison to diet model by van der Post & Hogeweg . . . 9

2 Reduction of traditional differences with increasing task difficulty 11

1 Detailed model description

(i) there is a diverse environment with different potential food or resource types of varying quality (Smuts et al., 1987; Chapman and Fedigan, 1990; Altmann, 1998); (ii) resources are limited in their abundance and are depleted when consumed, or contain aversive secondary compounds, such that foragers need to consume a variety of resources (Provenza, 1996); (iii) foragers live in groups with overlapping generations (Smuts et al., 1987); (iv) foragers need to learn about the rewards that are associated with particular resources (Matsuzawa and Hasegawa, 1982, 1983; Birch, 1999; Visalberghi et al., 2003; Hardus et al., 2014); (v) foragers are selective (i.e. do not eat everything they encounter) (Chapman and Fedigan, 1990; Altmann, 1998); (vi) foragers can develop skills associated with processing particular resources and so obtain greater rewards from those resources (Byrne, 1999; Jaeggi et al., 2010); (vii) foragers can observe the foraging choices of their neighbors and this can affect the foragers' subsequent choices (Visalberghi and Addessi, 2003; Fragaszy and Visalberghi, 2004; Agostini and Visalberghi, 2005; Jaeggi et al., 2010; van de Waal and Whiten, 2012; van de Waal et al., 2014). These empirical observations directly determine the main features of our model, and we study the dynamic processes that they generate. Here we document a full model description based on the ODD protocol for describing simulation models (Grimm et al., 2006).

1.1 Purpose

We develop an event-based, individual-based, spatially extended model of group foragers learning what and how to eat, in order to enable a multi-scale approach to the evolution of social learning mechanisms. Specifically, we study the cultural phenomena generated by evolved social learning mechanisms.

1.2 State variables and scales

Our model consists of a 2-dimensional environment with resource items and foragers. Space and time are continuous (via event-based scheduling), but some variables are updated via discrete time steps where each time point is a minute, a day is 720 minutes (only daylight), and a year is 360 days.

Resource types are described by: a spatial position, a maximum energy value Q_r, a task difficulty H_r and S_r which determines how rapidly foragers can initially learn to process a resource type. Specific resource items are also defined by a spatial position and whether they have been consumed this year or not. To generate resource patches, resource items are arranged randomly in a circle around a randomly selected patch center. Resource patches are characterized by: a location, a radius, resource density. The "patch-level" is emergent in the sense that patches can only be observed by foragers by sensing resource items.

Foragers are characterized by the following state variables: age, energy, position, heading, behavior state, behavioral action, time to complete an action (completion time), memory (see below), having a particular feeding target (i.e. a food item), a group identity, and parameters that determine behavior (see Table S1). Forager memory includes: (i) having a particular feeding target (i.e. a resource item), (ii) associations a_{ir} between a resource and a reward, (iii) experience t_{ir}, the total time spend processing a particular resource type, (iv) a particular resource type for

which the forager has a stimulus for via stimulus enhancement, (v) an estimate of the quality of resources that can be found in the environment a_{ie}, and (vi) estimates of how certain foragers are about resources c_{ir}. The forager population is fixed, but the number of groups changes when groups die and are 'born'.

1.3 Process overview and scheduling

We distinguish two levels of scheduling: (i) continuous time and (ii) discrete time steps (every minute).

Discrete updating: every minute we update individual age, reduce energy due to energy metabolism, determine if an individual dies due to a fixed death rate, starvation or old age, and if so select a parent according to energy levels:

$$({h_i}^W/(\sum_{j=1}^{N} h_j))^W \tag{S1}$$

where h_i is an individuals energy level, N is the population size and $W(=3)$ is the selection coefficient. Groups die when the last group member dies, and are born when a group reaches the maximum group size (20 foragers) and is randomly split into two equally sized daughter groups. Mutations in behavior parameters during reproduction generates genetic variation. If variation affects reproductive success, natural selection arises. Each resource type is checked if it is replaced by a new resource type every minute according to EC the rate of environmental change. Every DIG_TIME (100) minutes all consumed resource items are 'digested'. If any resource type exceeds SATIATION_FRAC*MAX_STOMACH number of items, then the forager attains a temporary satiation aversion for that resource type for SATIATION_TIME minutes.

Continuous updating: every behavioral action has a continuous time point at which it is completed (completion time). Actions can therefore end anywhere within a minute (with the resolution of "float" types in C++ programming language), and an action started in a previous minute is completed in the next minute. All foragers are put into a queue according to their completion time. The individual with the lowest completion time is next to complete its action and choose a new action. The completion time represents the duration of an action. Foragers therefore start their actions asynchronously, but action durations can overlap. This is an event-based scheduling. The sequence of actions of a forager is determined by the decision making algorithm.

When foragers consume resource items those items are tagged as 'consumed' and become available again the following year. When assessing resources items locally, any consumed resources items are checked whether they have become available again. If so they are tagged as available. This set-up was chosen for computational reasons, such that updating resource items occurs in a 'need to know' manner in relation to what foragers are observing locally. As a result we do not need to update all resource items all the time.

1.4 Design concepts

Emergence: our model is all about detecting and understanding emerging processes and the interactions between them. The following is a list of emerging phenomena:

Name	Description	Values
DAY	minutes of daylight foraging time	720
YEAR	number of days	360
PATCH_RADIUS	Radius of the patch	10 m
PATCH_RES	Number of resource types in a given patch type	5
PATCH_RES_SEL	Subset of possible resource types in particular patch	3
R	Number of resources species	250
N	Population size	100
W	Selection coefficient which determines the strength of selection	3
MAX_AGE	Maximum age that foragers can reach	20 years
DEATH_RATE	Rate of stochastic death	0.1 per year
BIRTH_ENERGY	Energy invested in offspring	5000 units
DIGESTIONTIME	Time interval at which digestion occurs	100 minutes
STOMACH_CAP	Number of items that can be eaten before forager is full	20
REACH	Distance at which forager can reach a resource item	0.9 m
M	The maximum event time (for EAT, OBSERVE, MOVE)	1 min
COPY_SPACE	Distance at which foragers can observe what their neighbors are doing	20
K	The proportion of the difference in experience that can be learning in skill learning by observation	0.1
SEARCH_ANGLE	Angle about forward heading that the forager searches	$\pi/2$
SEARCH_DISTANCE	Maximum distance at which resource items can be detected	2 m
NUM_ITEMS_PER_SEARCH	Number of items that can be assessed in SEARCH_TIME	20
MOVE_SPEED	Speed when moving forward	3.6 km / h
STEP_DISTANCE	Distance of a single move forward event	1 m
NOTHING_TIME	Duration of NOTHING action	M
EAT_TIME	Duration of EAT action	M
A	Area of the environment	39.2 km^2
DENSITY	Total resource density	0.75
PATCH_ITEMS	Number of resource items in patch	1200
PATCHES	Number of resource patches	24500
Q_r	Maximum energy reward of resource type r	$N(0.1, 0.1)$
H_r	Practice time needed before obtaining half the maximal reward	0.1..10
S_r	Scalar for the sigmoid function describing how rewards increase with practice	1..4
Z	Noise on the reward foragers obtain from a resource: standard deviation of a normal distribution about e_{ir}	0.005
EC_1	Rate at which resource species are replaced by new unknown species	0..R species per year
EC_2	Rate at which resource species change their quality Q_r	0..R species per year
METABOLISM	Energy used for metabolism	0.01 per min
SATIATION_TIME	Time that satiation lasts	DIGESTIONTIME
SATIATION_FRAC	Proportion of stomach filled by a resource type in order to become satiated	0.9
G	Number of foragers in a group	20
SAFE_NEIGHS	Number of neighbors a forager needs to feel safe	9
SAFE_SPACE	SAFE_NEIGHS should be within this distance	17 m
PROB_CHECK_SAFE	Probability to check if safe	0.9
ALIGN_PROB	Probability to align to neighs direction after MOVETOGROUP because not safe	0.9
ALIGN_ZONE	Zone of alignment	20 m
MOVETOGROUP_DISTANCE	Distance individual moves to become safe	3 m
MOVETOGROUP_SPEED	Speed at which forager move to become safe	180 m/min
SEARCH_TIME	Duration of SEARCH action	$0.5 * M$
δ_i	Distance foragers move forward after not finding preferred food	0..20 m
σ_i	Selectivity scalar	0..20
ϕ_i	Rate scalar for updating the expected reward for the environment a_{ie}	0..1
λ_i	Rate scalar for reinforcement learning	0..1
ε_i	Maximum exploration rate	0..1
γ_i	Increment in probability to select a resource if previously stimulated in stimulus enhancement	0..1
ω_i	Probability to OBSERVE neighbor	0..1
τ_i	Maximum time observing neighbor	0.01..1 min
e_{ir}	Reward obtained by forager i from resource r	0..$max(Q_r)$
h_i	Forager's total energy (or health)	0..∞
t_{ir}	Total time forager i has practiced processing resource r	0..MAX_AGE
a_{ir}	Reward forager i expects from resource r	0..$max(Q_r)$
a_{ie}	Reward forager i expects from environment	0..$max(Q_r)$
c_{ir}	Certainty estimate of expected reward	0..1
p_i	The time forager i has left to complete performing its present behavioral action	0..M
o_{ik}	Effective observation time by forager i of neighbor k equals $min(\tau_i, p_k)$	0..M

Table S1: List of parameters and variables. Upper case letters and names: to refer to invariant parameters that do not change during simulations. Lower case letters: variables of foragers that change during their lifetime. Greek letters: parameters that evolved. Subscripts: i = forager identity; r = resource type; k = forager's neighbor; e = environment.

- Foraging patterns: larger-scale detection of resource patterns beyond the local perception of individuals (van der Post and Semmann, 2011a,b).
- Patterns of resource depletion (van der Post and Hogeweg, 2006).
- Interactions between grouping and resource patterns (van der Post and Hogeweg, 2006).
- Learning patterns (van der Post and Hogeweg, 2006).
- Cultural phenomena (van der Post and Hogeweg, 2008; van der Post and Hogeweg, 2009).

Adaptation: individuals have a decision making algorithm that determines how they respond to the local environment.

Fitness: we define that the rate of reproduction of foragers depends on energy intake, therefore more feeding generates a greater birth rate. A forager's lifespan depends on a fixed death rate, starvation and maximum age.

Prediction: individuals do not predict future conditions, but respond to environmental conditions they encounter. These responses are embedded, in the sense that they are determined by locally observable conditions. We refer to this type of responsiveness as TODO, as in individuals DO WHAT THERE IS TO DO (Hogeweg and Hesper, 1985). What individuals do is determined by evolvable decision making algorithms.

Sensing: central for TODO is local information processing, leading to larger scale TODO-based pattern recognition beyond the direct perception of the individual (Hogeweg and Hesper, 1991; van der Post and Semmann, 2011a). We only define local perception of food (2 meters), and on a somewhat larger scale the awareness of neighbors.

Interaction: foragers can approach, eat and deplete resources. Foragers can detect each other and approach, align or move away from other individuals.

Collectives: resources patches and groups of foragers.

Observation: we collect data on what foragers eat, what their associations are, their experience, their group members, their age, energy, location, number of offspring, and evolvable parameters. Analysis generally proceeds via macro-observables (e.g. energy level) and becomes increasingly detailed as more information is needed to understand what and how processes are occurring (see main text).

1.5 Specification choices of scaling context

The scaling context of our model is determined by fixed parameters (Table S1):

Spatial and temporal resolution: At the minimal level we define the minimal time and spatial scale. Evolvable action durations can be minimally 10 seconds (t_{MIN}), to keep the model running fast enough. Space is continuous: individuals and resources can be placed at any given location.

Local information processing: Constraints on movement and perception (local information processing) are defined in terms of the spatial and temporal scale. Maximum speed is defined in terms of the minimal time interval, so that the duration of moving 1 meter can never be below t_{MIN}. The maximum range of resource detection (r_D) is then chosen to be small, so that individuals have to move to detect food (Video 1, gray semi-circle). Within this range all

resource items are detected. The individual's REACH d_r is then chosen to be below maximal resource detection range (Video 1, gray small circle). This is quite a reasonable assumption (equal detection range and REACH is probably a limit case).

For grouping we assume that groups are cohesive and assume that forager's can always keep track of all their group-mates, but ignore foragers of other groups. Moreover, for the alignment zone (ALIGN_ZONE) we assume a shorter range because alignment requires identifying the direction neighbors are moving.

Energy and life history: We fix the population at 100, and when a forager dies a parent is chosen according to its energy level E_i. The parent gives birth with cost E_B. This generates a viability threshold for the population, i.e. energy intake must be sufficient to have a net positive energy balance *and* must include sufficient energy for reproduction. If not, average energy levels drop and average energy levels in the population drop below E_B, in which case the population is not viable. In our simulations however, births and deaths continue.

Environment: The environmental settings were chosen to support selective foraging. This means that the resource density is so high that foragers do not end up eating everything, and need to be selective to reach the highest energy levels.

1.6 Initialization

We initialize the environment with a fixed number of resource types and multiple items of each type and patches with multiple resource types. An initial population of 100 foragers is initialized with parameters that have evolved. The population is initialized with 10 groups with 10 foragers each. Groups are initialized at random positions, where foragers start without an action, and with no information about the environment. Their first action will be FOODSCAN.

1.7 Input

The model does not include any external model or data files of driving environmental variables.

1.8 Submodels

Here we present a "mathematical" skeleton of the model updating schedule:

1.8.1 Forager continuous updates and decision making algorithm

During each minute:
while any action ends this minute, take soonest ending action:
1) Complete Action
——* MOVE: get new position
——* FOODSCAN: get new food target, or not
——* MOVETOFOOD: get new position
——* MOVETOGROUP: get new position
——* EAT: gain energy $E_i = E_i + e_{ir}$ and gain experience: t_{ir} and a_{ir}
where E_i is individual energy and e_{ir} is energy per food item
——* OBSERVE: gain experience t_{ir}

——* NOTHING: ready for next action
2) Choose New Action based on previous action:
—- if was MOVE: reselect MOVE if not yet reached δ_i meters
—- if NO ACTION YET ASSIGNED
——* CHECK IF SAFE and update any stimulus enhancement
——* if (not safe) then MOVETOGROUP
——* else (safe)
———** if was MOVETOGROUP then align to the direction of group members in ALIGN_ZONE
———** if was SEARCH or MOVETOFOOD then
—————-*** if resource target still exists
——————-**** if (distance to target $< d_R$)
———————-***** EAT
——————-**** else
———————-***** MOVETOFOOD
—————*** give up (food is gone)
———** if no action assigned and (RAND $< \omega_i$) then OBSERVE
———** else (no action and not observe)
—————*** if (stomach is full)
——————-**** do NOTHING
—————*** else
——————-**** if (previous action != FOODSCAN) and not crowded
————————***** FOODSCAN
——————-**** else
————————***** MOVE
3) Get new completion time
4) Place forager in action cue sorted by completion time.

RAND is a random number between 0 and 1, ω_i is the probability to OBSERVE, and d_R is the individual's reach.

1.8.2 Behavior actions of foragers

MOVE:

- Get new position and make sure it is on the grid:
  - $\vec{p'} = d_M\vec{v} + \vec{p}$
    where d_M is the MOVE distance, $\vec{v}$ = normalized random vector, and $\vec{p}$ is the position of the forager.
  - while $\bar{(}p'_x \; or \; p'_y < 0$ or $p'_x \; or \; p'_y >= \sqrt{A})$
    - $\vec{v}$ select new $\vec{v}$ (normalized random vector)
    - $\vec{p'} = d_M\vec{v} + \vec{p}$

    where A is the area of the environment.

- Duration: $d_M * t_M$
  where t_M is the time it takes the forager to move 1 meter.

MOVETOGROUP:

- $\vec{v} = \frac{\sum_{j \neq i}^{n_A} \vec{p}_j}{n_A}$
  n_A is the number of group members and $\vec{p}_j$ is the position of neighbor j.
- $\vec{p'} = d_M \vec{v} + \vec{p}$
  where d_M is the MOVE distance and $\vec{p}$ is the position of the forager.
- Duration: $d_M * t_M$
  where t_M is the time it takes the forager to move 1 meter, d_M is the step-distance.

FOODSCAN (Video 1, gray semi-circle): try to get new food target

- for all n food items (distance $< d_F$ and angle $< a_F/2$ from heading)
  where d_F is the radius of search, and a_F is the search angle about the foragers forward heading.
  - assess a maximum of 20 items
  - for each item decide whether to eat of not based on Equation 1.
- Duration: t_F

MOVETOFOOD: get new position closer to food item

- $\vec{v} = \frac{\vec{f} - \vec{p}}{|\vec{f} - \vec{p}|}$
- $d =$ distance to food where $\vec{f}$ is the position of food item and $\vec{p}$ the position of the forager
- if $d > d_r$
  $\vec{p'} = d_M \vec{v} + \vec{p}$
  else
  $\vec{p'} = d\vec{v} + \vec{p}$
- Duration: distance moved * t_M

EAT: consume food item

- Duration: t_E

OBSERVE: observe neighbor

- Duration: τ_i
- leads to acquisition of experience based on Equation 8.

NOTHING: do nothing

- Duration: t_N

1.8.3 Forager discrete updates

Each minute for all foragers:

- Age: age + 1 minute
- Energy: $E_i = E_i - E_m$
  where E_m is energy metabolism per minute
- Stimulus enhancement time (after stimulus):
  - if (stimulus duration < 30 minutes): stimulus time = stimulus time + 1 minute
  - if (stimulus duration >= 30) no more stimulus
- Reproduction:
  - if (population < 100): select parent according to energy (see Equation S1)
    * Energy change due to birth: $E_i = E_i - E_B$, where E_B is the birth cost.
    * Offspring energy: E_B
- Death (forager dies if):
  - $E_i \leq 0$
  - age >= maximum age
  - RAND < death rate (equivalent to 0.1 per year)

1.8.4 Resources

For all depleted resource items every year:

- all depleted resource items re-appear (or re-grow).
- resource items that are eaten disappear immediately.

This is not computed by looping over all items all the time, but only when those items are assessed by foragers locally.

1.9 Comparison to diet model by van der Post & Hogeweg

The present model differs from that of van der Post and Hogeweg (2006) in the following ways: (i) Here foragers can immediately sense rewards, while in van der Post and Hogeweg (2006) this was delayed to after digestion, enabling sensing of rewards to represent a mixture of resources. The latter makes learning complicated. This is not considered here in order to focus on skill learning, but in principle both skill learning and mixing of rewards could occur at the same time. (ii) In the present study we add skill learning and rewards increase of time. In van der Post and Hogeweg (2006) rewards were constant, but assessments thereof would develop from zero. (iii) To enable foragers to try unknown and less known resources, we implement an intrinsic

exploration tendency that depends on the forager's certainty estimate. In van der Post and Hogeweg (2006) this was implicit by assuming that individuals only explore resources for which they have no existing preference or association with a probability depending on 'trail rate'. The latter could effectively limit exploration via a familiarity-selectivity feedback. This could generate unfair implicit constraints on asocial learning, relative to which an advantage of social learning would be an artifact. Hence we added a more general exploration tendency, mitigated by knowledge of a resource ('certainty', see Equation 2).

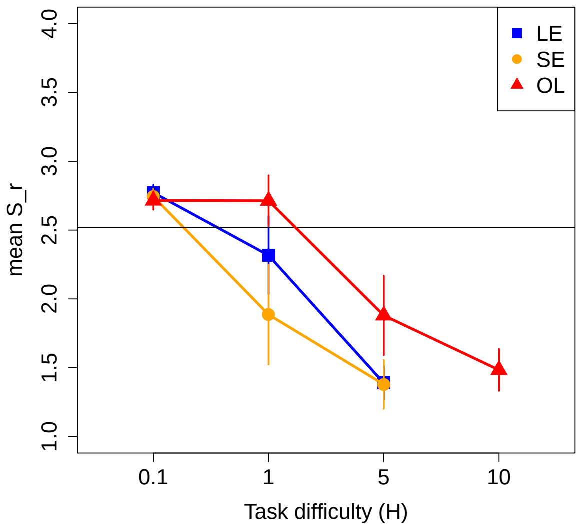

Figure S1: Mean S_r of consumed resources as a function of task difficulty (H). Blue = LE, Orange = SE, and Red = OL. LE and SE are inviable at H10 and are excluded. Each data point is the mean of 10 simulations, and whiskers show standard deviation.

2 Reduction of traditional differences with increasing task difficulty

Here we explain why the magnitude of traditional differences declines as task difficulty increases beyond H=1 (Fig. 2A). This patterns appear to be explained by a similar pattern of between-group differences declining once $H > 1$ (Fig. 3A). We therefore need to explain why between-group differences decline once $H > 1$.

Between-group differences arise when there are limits on population-wide convergence on particular subsets of resource types. For a given simulation, resources in our model only vary with respect to two characteristics: (i) resource quality Q_r and (ii) the rate at which rewards increase with practice defined by S_r (Equation 4, Fig 1C). Population-wide convergence on high quality resources is expected through the process of optimization. Convergence on particular values of S_r was not expected a priori, but is a possibility.

Our results show that as task difficulty increases, optimization expressed in terms of repertoire quality, becomes more limited (Fig 3C). As a result we would expect the scope for between-group differences to increase as H increases, since population-wide convergence on an optimum was becoming more limited. This implies that convergence on high quality resources is not the cause for a decline in between-group differences when $H > 1$. Thus we focus on S_r.

In Fig. S1 we show the mean value of S_r of consumed resources for different levels of task difficulty (H). Any convergence on extreme values of S_r will be revealed if the mean of S_r if either high (close to 4) or low (close to 1). The mean S_r value is indicated by the horizontal

gray line. We can observe that as H increases, the mean value of S_r declines and approaches one, implying a population-wide convergence on resource types with low S_r.

Low S_r means that for the same quality resource and practice time, foragers obtain a greater reward, than for resource types with high S_r. As a result, there is a stronger feedback between learning and preference, and stronger familiarity biases towards resources with low S_r. Thus as task difficulty increases, there is an increasing bias to the subset of resources that is easiest to learn.

References

Agostini, I. and E. Visalberghi, 2005. Social influences on the acquisition of sex-typical foraging patterns by juveniles in a group of wild tufted capuchin monkeys (Cebus nigritus). *Am J Primatol* **65**:335–51.

Altmann, S. A., 1998. Foraging for survival. The University of Chicago Press, Chicago.

Birch, L. L., 1999. Development of food preferences. *Ann. Rev. Nutr.* **19**:41–62.

Byrne, R., 1999. Cognition in great ape ecology: skill-learning ability opens up foraging opportunities. In H. O. Box and K. R. Gibson, editors, *Mammalian social learning: Comparative and ecological perspectives*, pages 333–350, Cambridge, UK. Cambridge University Press.

Chapman, C. A. and L. M. Fedigan, 1990. Dietary differences between neighboring *Cebus capucinus* groups: local traditions, food availability or responses to food profitability? *Folia Primatol* **54**:177–86.

Fragaszy, D. and E. Visalberghi, 2004. Socially biased learning in monkeys. *Learn Behav* **32**:24–35.

Grimm, V., U. Berger, F. Bastiansen, S. Eliassen, V. Ginot, J. Giske, J. Goss-Custard, T. Grand, S. Heinz, G. Huse, et al., 2006. A standard protocol for describing individual-based and agent-based models. *Ecological modelling* **198**:115–126.

Hardus, M. E., A. R. Lameira, S. A. Wich, H. de Vries, R. Wahyudi, R. W. Shumaker, and S. B. J. Menken, 2014. Effect of repeated exposures and sociality on novel food acceptance and consumption by orangutans. *Primates* .

Hogeweg, P. and B. Hesper, 1985. Socioinformatic processes: MIRROR modelling methodology. *J. Theor. Biol.* **113**:311–330.

Hogeweg, P. and B. Hesper, 1991. Evolution as pattern processing: TODO as substrate for evolution. In J.-A. Meyer and S. W. Wilson, editors, *Proceedings of the first international conference on simulation of adaptive behavior on From animals to animats*, pages 492–497. MIT Press, Cambridge, Mass.

Jaeggi, A. V., L. P. Dunkel, M. A. V. Noordwijk, S. A. Wich, A. A. L. Sura, and C. P. V. Schaik, 2010. Social learning of diet and foraging skills by wild immature Bornean orangutans: implications for culture. *Am. J. Primatol.* **72**:62–71.

Matsuzawa, T. and Y. Hasegawa, 1982. Food-aversion conditioning in Japanese monkeys (Macaca fuscata): suppression of key-pressing. *Behav Neural Biol* **36**:298–303.

Matsuzawa, T. and Y. Hasegawa, 1983. Food aversion learning in Japanese monkeys (*Macaca fuscata*). A strategy to avoid a noxious food. *Folia Primatol* **40**:247–255.

Provenza, F. D., 1996. Acquired aversions as the basis for varied diets of ruminants foraging on rangelands. *J. Anim. Sci.* **74**:2010–2020. Diet development.

Smuts, B. B., D. L. Cheney, R. M. Seyfarth, R. W. Wrangham, and T. T. Struhsaker, 1987. Primate Societies. The University of Chicago Press, Chicago.

van de Waal, E., N. Claidière, and A. Whiten, 2014. Wild vervet monkeys copy alternative methods for opening an artificial fruit. *Anim Cogn* .

van de Waal, E. and A. Whiten, 2012. Spontaneous emergence, imitation and spread of alternative foraging techniques among groups of vervet monkeys. *PLoS ONE* **7**:e47008.

van der Post, D. J. and P. Hogeweg, 2006. Resource distributions and diet development by trial-and-error learning. *Behav. Ecol. Sociobiol.* **61**:65–80.

van der Post, D. J. and P. Hogeweg, 2008. Diet traditions and cumulative cultural processes as side-effects of grouping. *Anim. Behav.* **75**:133–144.

van der Post, D. J. and P. Hogeweg, 2009. Cultural inheritance and diversification of diet in variable environments. *Anim. Behav.* **78**:155–166.

van der Post, D. J. and D. Semmann, 2011a. Local orientation and the evolution of foraging: Changes in decision making can eliminate evolutionary trade-offs. *PLoS Comput. Biol.* **7**:e1002186.

van der Post, D. J. and D. Semmann, 2011b. Patch depletion, niche structuring and the evolution of cooperative foraging. *BMC Evol. Biol.* **11**:335.

Visalberghi, E. and E. Addessi, 2003. Food for thought: social learning about food in capuchin monkeys, in: D.M. Fragaszy and S. Perry (eds) The Biology of Traditons, pages 187–212. Cambridge University Press.

Visalberghi, E., G. Sabbatini, M. Stammati, and E. Addessi, 2003. Preferences towards novel foods in Cebus apella: the role of nutrients and social influences. *Physiology and Behavior* **80**:341–349. dio:10.1016/j.physbeh.2003.08.004.
